# A DUTY TO CARE: MALE PERSPECTIVES ON THE CAREGIVER ROLE FOR PERSONS WITH ALZHEIMER’S OR RELATED DEMENTIA

**DOI:** 10.1093/geroni/igac059.397

**Published:** 2022-12-20

**Authors:** Michael Bueno, Jo-Ana Chase

**Affiliations:** University of California, Irvine, Placentia, California, United States; University of Missouri, Columbia, Missouri, United States

## Abstract

The population of family caregivers (FCGs) of persons with Alzheimer's Disease and related Dementias (ADRD) is growing, as is the proportion of males taking on this traditionally female role. Most caregiving research has mainly focused on females. Although female caregivers have reported more negative outcomes, men still report significant levels of burden. With the aging population and increased need for caregivers, there is a gap in knowledge exploring the male caregiving experience. Understanding male caregiving experiences can inform clinicians on developing future strategies to tailor support for this underrepresented group. The purpose of this qualitative descriptive study was to explore the experiences of male FCGs of people with ADRD. The Caregiver Identity Theory (CIT) was used to guide the study exploring participants’ perception of self-identity within their caregiving relationship and self-identity as a male. Eleven male caregivers, recruited through social media and community resources, were interviewed by telephone or Zoom. Interviews were recorded, transcribed, and analyzed using thematic analysis. Four major themes emerged highlighting males’ struggles with the unfamiliar caregiving role and changing identity; their acknowledgement of personal growth and discovery through caregiving, challenges in finding the “right” kind of support, and perceived reshaping of masculinity through the caregiving role. Male caregivers express unique experiences as FCGs suggesting future research is needed to explain gender differences in caregiving and identify additional factors that influence male caregivers’ experiences. Furthermore, findings indicate clinicians should tailor support strategies for male FCGs’ as they fulfill this potentially unfamiliar role.

